# To the Editors of the Medical and Physical Journal

**Published:** 1799-06

**Authors:** Charles Brown

**Affiliations:** Hatton Garden


### *To the Editors of the Medical and Phyſical Journal.*

GENTLEMEN,

### *To the Editors of the Medical and Phyſical Journal.*

GENTLEMEN,

I Herewith ſend you the morbid appearances of a ſubject I opened laſt week: the particulars of his illneſs I am not acquainted with; I was informed by the phyſician who attended him, that he had laboured two years under ſpaſmodic aſthma, with a conſtant throbbing of the heart. Laſt Thurſday, while walking out of his houſe in Cavendiſh-ſquare, to get in his carriage, he dropped down, and expired immediately.

*Appearances*

*Appearances on Dissection.*

In the right ventricle of the heart, there was a mass of coagulable lymph, which extended into the neighbouring large vessels, very firm, and of a light yellow colour; it did not fill up the cavity completely. The apex of the left ventricle was dilated into a pouch, about the size of a large walnut. There was an aneurism at the arch of the aorta; the latter was enlarged beyond its usual size. The three semilunares were thickened and opaque. The coats of the pulmonary artery were elongated, and of a light reddish colour. The valvulæ mitrales were nearly obliterated. The lungs were full of tubercles, mostly in a state of suppuration. The liver was enlarged, and there were two calculi, very small, in the *ductus hepaticus*. The other viscera were perfectly sound.

Your obliged

*Hatton Garden,* CHARLES BROWN.

*May 10th,* 1799.

P. S. I am under the necessity of deferring my paper on the antivenereal virtues of the *hydrargyrum phosphoratum*, and my experiments with the *yellow oxyd of tungsten*, till I have leisure to complete both.

